# A neurotrophic withdrawal hypothesis for multisystem adverse events associated with anti-NGF therapy for canine osteoarthritis pain

**DOI:** 10.3389/fvets.2026.1776920

**Published:** 2026-09-11

**Authors:** Giovanni Federico, Alfonso Ilardi, Valerio Elia, Sergio Chieffi

**Affiliations:** 1 Department of Education, Psychology and Communication, Suor Orsola Benincasa University, Naples, Italy; 2 Inmates Ward, Department of Internal Medicine, Antonio Cardarelli Hospital, Naples, Italy; 3 Department of Experimental Medicine, University of Campania “Luigi Vanvitelli”, Naples, Italy

**Keywords:** adverse events, bedinvetmab, izenivetmab, Lenivia, Librela, monoclonal antibodies, NGF

## Abstract

Post-marketing reports in dogs treated with *bedinvetmab* (Librela™, Zoetis), the first anti-nerve growth factor (NGF) monoclonal antibody approved for canine osteoarthritis pain, include musculoskeletal, autonomic, and neurological events. Existing interpretations address these domains separately or focus on the limitations of pharmacovigilance systems. Here, we propose a complementary, integrative interpretation: at least some of these events may share an on-target component arising from systemic NGF sequestration—an iatrogenic state of neurotrophic withdrawal. The evidence derives mainly from *bedinvetmab*, but the recent authorization of a second canine anti-NGF agent, *izenivetmab* (Lenivia™, Zoetis), raises a testable class-level safety question. Drawing on neurotrophin biology, human anti-NGF development programs, regulatory data, and the most recent evidence linking somatosensory innervation to skeletal repair, we outline how reduced NGF-mediated trophic support might affect joint protection and load adaptation/fracture repair, autonomic regulation, and central neuronal maintenance, particularly in geriatric animals. We set out the predictions that distinguish this account from unrelated background disease, and the studies that would test them.

## Introduction

Nerve growth factor (NGF), the prototypic neurotrophin, exerts pleiotropic effects on the survival, plasticity, and homeostatic regulation of multiple neuronal populations by signaling through tropomyosin receptor kinase A (TrkA) and the p75 neurotrophin receptor (p75NTR) (1–4). Therefore, the therapeutic neutralization of NGF for chronic pain has long been recognized as a strategy whose effects may extend beyond nociception. In human medicine, for example, this concern became clinically evident during the development of *tanezumab*, a humanized anti-NGF monoclonal antibody developed for osteoarthritis pain: dose-related rapidly progressive osteoarthritis (RPOA) and other serious joint-related adverse events ultimately constrained the program despite analgesic efficacy (5, 6).

*Bedinvetmab* (Librela™, Zoetis), a caninized anti-NGF monoclonal antibody licensed for the treatment of canine osteoarthritis pain, has been associated with a heterogeneous post-marketing adverse-event signal. U.S. regulatory summaries include reports of urinary dysfunction, ataxia, paresis, lethargy, behavioral change or disorientation, impaired consciousness, and seizures (7, 8). Peer-reviewed analyses and regulatory assessments, including a specialist-led review of EudraVigilance reports and a clinical case series (9), have raised parallel concerns regarding musculoskeletal abnormalities (9–11).

The heterogeneity of these events has prompted divergent interpretations, centered either on RPOA (11) or on limitations of pharmacovigilance systems that may obscure or distort the underlying signal (12). The 2026 Veterinary Medicines Directorate (VMD) assessment concluded that the overall benefit–risk balance remained positive, while noting that causal attribution remained unclear for several evaluated outcomes and that continued surveillance was required (10). However, this population-level regulatory determination does not test whether an on-target mechanism contributes to a subset of reported events.

In this Perspective, we argue that the multisystem adverse-event pattern is biologically consistent with iatrogenic neurotrophic withdrawal following systemic NGF sequestration. The hypothesis predicts that geriatric, multimorbid dogs with limited capacity to compensate for reduced trophic signaling would be especially vulnerable. We set out the receptor biology underlying this hypothesis, examine how a shared on-target contribution could link selected musculoskeletal and urinary or autonomic events and, more tentatively, central neurological events (summarized in Table 1), and specify the evidence needed to test it.

**Table 1:** Multisystem adverse events reported in dogs treated with *bedinvetmab* and their proposed relationship to neurotrophic withdrawal.

Clinical event	Proposed mechanism	Potentially relevant systems
Ataxia, paresis, and proprioceptive deficits	Joint instability may produce pseudo-ataxia, and limb collapse under load may mimic paresis; reduced nociceptive input and altered dorsal horn processing may weaken protective motor responses. These mechanisms cannot explain confirmed proprioceptive deficits.	Nociceptive afferents; dorsal horn circuitry; musculoskeletal interface
Rapid joint collapse, fractures, and ligament ruptures	Anti-NGF therapy may create a dual-hit: analgesia can reduce protective unloading while withdrawal of NGF–TrkA signaling could weaken sensory feedback and the trophic support required for skeletal load adaptation and fracture repair.	Nociceptive afferents; sensory-neuronal support of joint and skeletal integrity
Urinary incontinence and altered voiding; polyuria and polydipsia	Reduced bladder afferent signaling and sympathetic support of the bladder neck may impair filling sensation and continence. Polyuria and polydipsia are physiologically distinct and require separate investigation.	Lower urinary tract afferents and post-ganglionic sympathetic neurons (for incontinence and altered voiding)
Apathy, lethargy, and cognitive dulling	Peripheral NGF sequestration and age-related BBB vulnerability could reduce central trophic support (NGF efflux via a peripheral sink), potentially promoting cholinergic hypofunction and neurodegeneration.	Basal forebrain cholinergic system
Seizures	If central NGF homeostasis were altered, indirect basal forebrain-cortical effects might modify seizure susceptibility in vulnerable dogs.	Basal forebrain cholinergic projections and cortical networks (indirect and hypothetical)

## NGF signaling

NGF signals primarily through TrkA and p75NTR, whose effects depend on ligand form and receptor context. The binding of mature NGF to TrkA sustains trophic signaling, neuronal phenotype, and plasticity, while p75NTR can facilitate TrkA signaling (3, 4, 13, 14). By contrast, binding of proNGF to a p75NTR-sortilin complex can activate apoptotic and degenerative pathways (15, 16). ProNGF has been reported to predominate in adult mammalian brain preparations (16–18), and aging can impair TrkA expression, signaling, and retrograde transport (18, 19). Systemic NGF neutralization might therefore weaken an already compromised trophic axis and expose vulnerable neurons to degenerative signaling. Whether therapeutic NGF neutralization produces this shift in canine neural tissue remains to be determined in future studies. To the extent that a given antibody binds mature NGF with substantially greater affinity than proNGF, a product-specific property that has not been systematically characterized for these agents, selective neutralization would be expected to increase the ratio of bioavailable proNGF to free mature NGF, all else being equal. This could alter the relative contribution of proNGF-dependent signaling in addition to reducing mature-NGF-dependent TrkA signaling. The prediction is directional and experimentally testable, and should be most relevant in peripheral compartments in which antibody and ligand coexist, including peripheral target tissues and, plausibly, dorsal root ganglia.

This receptor biology provides the mechanistic basis for a unified account of the *bedinvetmab* safety signal. The reported signs are functionally diverse, yet they involve systems containing NGF-responsive cells: subsets of nociceptive dorsal root ganglion neurons, post-ganglionic sympathetic neurons, lower urinary tract afferents, and basal forebrain cholinergic neurons (1, 3, 20–26). Thus, systemic disruption of NGF signaling may offer a parsimonious working explanation for a multisystem phenotype without requiring a separate toxic mechanism for each event.

## Musculoskeletal manifestations: a “dual-hit” model

Ataxia, paresis, and proprioceptive deficits are among the events reported after *bedinvetmab* treatment (7, 8). Primary proprioceptive afferents depend predominantly on neurotrophin-3 signaling through tropomyosin receptor kinase C (TrkC) and are therefore not expected to be direct targets of NGF neutralization (2, 3). The clinical phenotype can nevertheless arise through converging musculoskeletal and neural mechanisms.

In humans, *tanezumab* reduced osteoarthritis pain (27); in a rat meniscal-tear model, it increased weight-bearing while worsening cartilage damage (28). Joint instability and compensatory gait changes may then mimic ataxia (*i.e*., “pseudo-ataxia”), and limb collapse under load can be misinterpreted as paresis. This reduction in protective nociception resembles the pathophysiological logic of Charcot neuro-osteoarthropathy, in which progressive joint destruction produces secondary gait disturbance (29). However, anti-NGF therapy produces only partial loss of nociceptive protection, rather than the more profound loss of protective sensation seen in classical neuropathic arthropathy. Because this protective loss is incomplete, the Charcot-like mechanism cannot, by itself, account for severe or progressive neurological signs and therefore cannot be used to rule out primary nervous-system disease. The analogy is thus mechanistic rather than diagnostic: it explains *how* musculoskeletal dysfunction may mimic neurological signs but does not license attributing such signs to mimicry in any individual case. Ataxia, paresis, and proprioceptive deficits should accordingly be treated as potentially neurological until dedicated assessment has excluded primary nervous-system disease.

Sustained NGF neutralization may also alter nociceptor phenotype, peripheral innervation, and dorsal horn plasticity, weakening the protective motor responses that normally limit loading of a damaged joint. Critically, the two components of this model are not independent. NGF–TrkA signaling in skeletal sensory nerves contributes to skeletal adaptation to mechanical loading: in mice, loading rapidly upregulates NGF expression in osteoblasts, and interruption of TrkA signaling markedly attenuates load-induced bone formation and reduces osteocytic Wnt/β-catenin activity (30). Anti-NGF therapy may therefore increase loading of a compromised joint while blunting a mechanism by which the skeleton adapts to that loading. This combination would define a “dual-hit” model: increased mechanical stress on the diseased joint combined with weakened sensory feedback and trophic support. Because this pathway is peripheral, the mechanism does not depend on antibody entry into the central nervous system.

The dual-hit model may help explain the fractures, ligament ruptures, and rapidly progressive joint changes reported in canine pharmacovigilance; these events parallel the RPOA observed in human *tanezumab* trials (5, 6, 9, 11). In those programs, the risk was not uniform: RPOA occurred considerably more often when anti-NGF treatment was combined with non-steroidal anti-inflammatory drugs (NSAIDs) than during monotherapy, and exclusion of chronic concomitant NSAID use became a formal risk-minimization measure (27, 31). Analgesia alone is not the distinctive risk, since potent analgesia may reduce protective unloading; the concern specific to anti-NGF therapy is that analgesia is superimposed on the withdrawal of trophic signaling. Thus, the clinical implication is not to withhold pain relief, but to avoid unmonitored restoration of activity, to carefully consider the potential risks of prolonged concomitant NSAID use, and to recognize accelerated joint compromise early (32).

Recent evidence in mice adds a second skeletal mechanism: somatosensory neurons that innervate bone release trophic and regenerative signals, including fibroblast growth factor 9 (FGF9), that support efficient fracture healing, whereas loss of sensory innervation severely impairs skeletal repair (33). FGF9-dependent signaling supports osteoprogenitor cell proliferation, osteogenesis, and angiogenesis, which are central components of repair (34). The bone-innervating neurons that supply these regenerative signals are predominantly calcitonin gene-related peptide (CGRP)-expressing afferents within the TrkA-dependent peptidergic population shaped by NGF (30, 33, 35). Pharmacological interruption of TrkA signaling is likewise sufficient to reduce sensory fiber density, blunt revascularization, and delay fracture-callus ossification (35). An earlier mouse study using neutralizing antibodies against NGF or TrkA did not find measurable impairment of fracture healing at the exposures tested (36), so clinical antibodies cannot be assumed to reproduce the effects of direct TrkA inhibition. Nevertheless, sustained anti-NGF therapy could compromise the sensory-neuronal trophic milieu that supports skeletal regeneration. This possibility has received very little attention in veterinary discussions and should be tested explicitly.

## Lower urinary tract manifestations

NGF regulates the development, maintenance, and plasticity of sympathetic and sensory innervation in the lower urinary tract (LUT), including the bladder and bladder neck (20–23). Many bladder afferents involved in nociception and mechanotransduction express TrkA, and inflammatory states increase urothelial NGF expression and afferent excitability (22, 24–26). Systemic NGF neutralization could therefore act on two timescales: a relatively rapid reduction in the excitability of TrkA-expressing bladder afferents and, with prolonged exposure, a reduction in trophic support for the sensory and sympathetic neurons supplying the bladder and bladder neck (22, 37). These two hypothetical mechanisms could produce different clinical outcomes—impaired filling sensation or incomplete voiding in the first case, and reduced urethral closure pressure in the second—but neither has been demonstrated after anti-NGF therapy.

Urinary incontinence has been reported following *bedinvetmab* exposure in post-marketing surveillance (7, 8). Attributing such events to treatment in an individual animal is nonetheless difficult, since incontinence in geriatric dogs is common and multifactorial. No validated biochemical surrogate resolves this: serum and urinary NGF concentrations did not differ according to the presence or absence of micturition dysfunction in dogs with spinal disease (38), and neither measure has been evaluated in dogs receiving anti-NGF antibodies. Attribution therefore rests on clinical observation and temporal association, and the requirement runs in both directions: voiding and continence events warrant monitoring as possible on-target effects on LUT afferent or autonomic pathways and should not be dismissed by default as consequences of aging.

Because owners may describe any change in urination as a single complaint, polyuria and polydipsia should be separated explicitly from incontinence: they are physiologically distinct and require independent renal, endocrine, metabolic, medication-related, and systemic evaluation.

## Central nervous system manifestations

Reports of lethargy, apathy, cognitive dulling, and seizures (7, 8) are difficult to reconcile with the limited blood–brain barrier (BBB) penetration of monoclonal antibodies. Nonetheless, three non-mutually exclusive mechanisms may account for such signs.

First, age-related BBB breakdown, including increased permeability, has been demonstrated in the human hippocampus (39), and age-related morphological changes have been described in the canine BBB [(40); see also (41)]. A recent magnetic resonance imaging study found changes suggestive of BBB dysfunction in dogs with suspected canine cognitive dysfunction, but no significant group-level difference from controls (42). Because many dogs treated with *bedinvetmab* are geriatric, age-related barrier compromise could increase the likelihood of central antibody exposure; whether such changes allow pharmacologically meaningful entry of an intact monoclonal antibody should be explicitly investigated in future studies. Moreover, although osteoarthritis is more common in older dogs, it also occurs in younger dogs (43), so this mechanism would apply only to a subset of cases.

Second, peripheral NGF sequestration may create a concentration gradient that draws NGF from the central compartment: a kind of “sink” phenomenon conceptually analogous to that proposed for β-amyloid (44). This analogy remains untested for NGF, remains contested within the amyloid literature from which it is borrowed, and is further limited by the fact that central NGF availability depends largely on local production and retrograde uptake rather than on exchange with a plasma pool. However, if central NGF availability were reduced, the consequences could be particularly severe for basal forebrain cholinergic neurons, which rely on NGF to maintain their phenotype and function, and to support attention, arousal, and cognitive processing.

Third, the aging mammalian brain shows dysmetabolism of the NGF pathway, with shifts in the mature NGF/proNGF balance and altered TrkA/p75NTR ratios. These changes have been proposed as central to cognitive decline, and canine cognitive dysfunction serves as a naturally occurring translational model (45). In this model, therapeutic NGF sequestration might accelerate an existing trajectory, with lethargy and apathy reflecting cholinergic hypofunction, and neurodegeneration with cortical network destabilization reducing the seizure threshold. This latter point has indirect experimental support: a proNGF/NGF imbalance has been associated with epileptiform activity in ex vivo preparations from transgenic mice (13); in humans, neurodegenerative disease and epilepsy are linked bidirectionally (46).

In sum, two routes could allow systemic NGF neutralization to affect central function: antibody passage across a compromised BBB, and a peripheral sink that lowers central NGF availability. Pre-existing NGF metabolic dysfunction may, in turn, determine which animals are vulnerable to either. Both routes are testable, and establishing whether either operates to a pharmacologically meaningful extent in treated dogs is a priority for future veterinary research.

## Discussion

Taken together, the reported musculoskeletal, urinary, and neurological events constitute a cross-domain safety signal warranting mechanistic investigation. We argue that neurotrophic withdrawal provides a parsimonious, testable framework for a possible on-target contribution because the proposed domains contain NGF-responsive elements. Importantly, this mapping does not establish that every report arose through that mechanism.

Notably, NGF exerts trophic and neuroprotective effects on a defined population of dorsal root ganglion (DRG) neurons. In adult rat L4/L5 DRG, TrkA-immunoreactive neurons comprise approximately 40% of cells and are overwhelmingly CGRP-positive (47). The same biological logic extends to sympathetic, lower urinary tract, and basal forebrain cholinergic systems.

This Perspective integrates evidence from the neurotrophin, skeletal-repair, and pharmacovigilance literature into a single biologically grounded, class-level safety hypothesis: neurotrophic withdrawal. Human experience with *tanezumab* provides an important clinical parallel (5, 6). The characteristics of the treated population further strengthen this concern: aged, comorbid dogs may have reduced TrkA signaling, compromised blood–brain barrier integrity, and limited neural reserve, and may therefore be less able to compensate for withdrawal of trophic support.

Our hypothesis does not require every treated dog to develop an adverse event, nor does it assume that every reported event is drug-induced. Susceptibility may vary with trophic reserve, receptor expression, BBB integrity, comorbidity, and pre-existing neurological or autonomic conditions. Additionally, spontaneous reports are subject to under-reporting, inconsistent coding, missing denominators, confounding, and stimulated reporting; these factors can dilute, amplify, or distort the observed signal (7, 12).

The main limitation of this Perspective's hypothesis is the lack of direct data in dogs; much of the supporting evidence comes from preclinical studies in other species and from human anti-NGF programs. Alternative explanations (*e.g*., the progression of osteoarthritis, age-related neurological disease, or unrelated comorbidities) remain plausible in individual dogs and may themselves contribute to a multisystem pattern; prospective comparative testing is therefore required.

Critically, the hypothesis we propose in this Perspective is falsifiable. Thus, prospective canine studies should use matched osteoarthritis comparators and serial neurological, orthopedic, cognitive, and urinary assessments, with exposure data and blinded event adjudication. Mechanistic studies should assess free and antibody-bound NGF, skeletal repair, and, where feasible, antibody exposure in cerebrospinal fluid or neural tissue. Pharmacovigilance requires harmonized terminology, transparent coding, suitable denominators, and independence from sponsor influence (12).

Although the available evidence discussed in this Perspective primarily relates to *bedinvetmab*, the recent authorization of another canine anti-NGF monoclonal antibody, *izenivetmab* (Lenivia™, Zoetis), raises an important class-level safety question. *Izenivetmab* is a caninized monoclonal antibody authorized for the reduction of osteoarthritis pain in dogs and administered at 3-month intervals. Its clinical efficacy has been evaluated in two exploratory dose-determination studies and one placebo-controlled field trial. The pivotal 9-month trial enrolled 574 client-owned dogs. At day 90, the model-estimated Canine Brief Pain Inventory (CBPI) treatment-success proportions were 36.3% with *izenivetmab* and 21.4% with saline (absolute difference: 14.9 percentage points; *P* = 0.0029). This adjusted difference corresponds to an approximate, timepoint-specific, trial-based number needed to treat of 6.7 for one additional CBPI-defined success at day 90 relative to saline; conversely, the model-estimated proportion not meeting the pre-specified success criterion with *izenivetmab* was 63.7%.

Osteoarthritis was confirmed radiographically at enrollment, but no serial joint imaging was reported. The public dossier, therefore, cannot determine whether analgesia altered structural disease progression—the safety outcome that proved consequential in human anti-NGF programs. Two further and distinct limitations apply: safety during concomitant NSAID administration has not been established, and effects beyond 9 months have not been evaluated. In the field trial, ataxia occurred in 8 of 289 treated dogs and in none of 285 controls. Plausible alternative explanations were identified for 5 of these 8 cases, but not for the remaining 3. These alternatives weaken attribution in individual cases but do not resolve the imbalance between treatment and control groups. Polydipsia occurred in 10 treated dogs and 2 controls, and polyuria occurred in 10 treated dogs and 1 control. Lethargy, the most frequently reported event, occurred in 39 treated dogs and 22 controls; although non-specific, it is consistent with the central component of the present hypothesis.

In a 26-week margin-of-safety study, minimal neuronal atrophy with increased glial cell density was observed in a single cranial mesenteric ganglion in each of 2 of 8 dogs given six times the maximum recommended dose. Of the 168 ganglia evaluated across all 24 izenivetmab-treated dogs, the other 166 showed no such findings. The findings were not associated with clinical signs. The regulatory assessment considered the overall safety margin adequate, and these localized supratherapeutic findings do not suggest neurotoxicity at clinical doses. However, their localization to an NGF-responsive sympathetic ganglion is mechanistically significant in the context of our hypothesis and may serve as a clear endpoint for targeted follow-up. Remarkably, the product literature notes that primates given high doses of anti-NGF monoclonal antibodies exhibited anatomical changes in post-ganglionic cell bodies, with reduced neuronal size and lower estimated neuron counts; the size change reportedly returned to control levels after discontinuation [(50); see also: (51)]. Thus, findings in two species involve the same broad neuronal class—postganglionic sympathetic neurons—supporting the need for prospective assessment of sympathetic structure and function rather than relying solely on clinical signs.

Taken together, the neurological and urinary imbalances and the ganglion findings neither establish causality nor prove a class effect. They do, however, show that the current evidence cannot exclude uncommon, delayed, or structurally mediated on-target harm. Hence, pre-specified long-term surveillance incorporating serial joint imaging and standardized assessments is required to determine whether these observations remain isolated or develop into a reproducible class pattern (48–50).

## Conclusion

Adverse events reported after *bedinvetmab* encompass neurological, musculoskeletal, and urinary signs. Although spontaneous reports cannot, by themselves, establish causation in individual dogs, demonstrate a shared mechanism, or estimate true incidence, this multisystem spectrum is biologically compatible with perturbation of NGF-responsive sensory and sympathetic pathways and warrants testing whether indirect mechanisms contribute to signs suggestive of central nervous system dysfunction. It therefore cautions against assuming that the physiological effects of anti-NGF monoclonal antibodies are restricted to peripheral nociception. The absence of reliable incidence estimates is not evidence that such events are rare; it is itself a major unresolved gap in the safety evidence.

Pending independent long-term data on incidence, susceptibility, cumulative risk, and reversibility, a cautious, individualized approach to *bedinvetmab* use appears warranted. This positioning follows from a precautionary judgment under uncertainty and not from the hypothesis itself, which is not evidentially sufficient to support a clinical recommendation on its own. *Bedinvetmab*, in our view, should be reserved for dogs with otherwise uncontrolled pain or unacceptable quality of life after established multimodal strategies have failed, are contraindicated, or are poorly tolerated. Similar caution may be appropriate for other anti-NGF agents, including *izenivetmab*, pending separate evaluation of each product. Longer dosing intervals strengthen the case for restraint because, once administered, exposure cannot be promptly curtailed. The relevant evidence for reversibility comes from non-human primate studies of *tanezumab*, in which non-progressive morphological changes in sympathetic ganglia, unaccompanied by neuronal death or detected sympathetic dysfunction, reversed during a non-dosing recovery period (51). Reversibility characterized in this way is nonetheless conditional on dosing that can be interrupted. This precautionary position should be weighed against the harms of undertreated pain (32) and does not imply an unfavorable benefit–risk balance in every patient.

Before treatment, clinicians should obtain documented informed consent, record baseline neurological and orthopedic status, and assess renal and urinary function where clinically indicated. Use should be conditional on a graded return to activity, predefined stopping criteria, reassessment before every dose, and active surveillance for neurological, musculoskeletal, and urinary abnormalities, with renal monitoring as appropriate. Selective use, structured monitoring, and transparent post-authorization pharmacovigilance are, in our view, the minimum proportionate safeguards for prolonged systemic neutralization of a pleiotropic neurotrophin.

## References

[1] Sofroniew MV Howe CL Mobley WC. Nerve growth factor signaling, neuroprotection, and neural repair. Annu Rev Neurosci. (2001) 24:1217–81. doi: 10.1146/annurev.neuro.24.1.1217 11520933

[2] Tessarollo L. Pleiotropic functions of neurotrophins in development. Cytokine Growth Factor Rev. (1998) 9:125–37. doi: 10.1016/S1359-6101(98)00003-3 9754707

[3] Reichardt LF. Neurotrophin-regulated signalling pathways. Philos Trans R Soc Lond B Biol Sci. (2006) 361:1545–64. doi: 10.1098/rstb.2006.1894 16939974 PMC1664664

[4] Chao MV. Neurotrophins and their receptors: a convergence point for many signalling pathways. Nat Rev Neurosci. (2003) 4:299–309. doi: 10.1038/nrn1078 12671646

[5] Hochberg MC Tive LA Abramson SB Vignon E Verburg KM West CR . When is osteonecrosis not osteonecrosis? Adjudication of reported serious adverse joint events in the tanezumab clinical development program. Arthritis Rheumatol. (2016) 68:382–91. doi: 10.1002/art.39492 26554876

[6] Miller RE Malfait AM Block JA. Current status of nerve growth factor antibodies for the treatment of osteoarthritis pain. Clin Exp Rheumatol. (2017) 35(Suppl 107):S85–S87. 28967370 PMC6007861

[7] Smith J Hugunin K. Standard Adverse Event Pharmacovigilance Review for Librela™ *(Bedinvetmab Injection), NADA 141-562 (DVPS-2024-141)*. U.S. Food and Drug Administration, Center for Veterinary Medicine (2024). Available online at: https://www.fda.gov/media/184483/download (Accessed July 19, 2026).

[8] U.S. Food and Drug Administration (FDA). Dear Veterinarian Letter Notifying Veterinarians About Adverse Events Reported in Dogs Treated with Librela (Bedinvetmab Injection) (2024). Available online at: https://www.fda.gov/animal-veterinary/product-safety-information/dear-veterinarian-letter-notifying-veterinarians-about-adverse-events-reported-dogs-treated-librela (Accessed July 19, 2026).

[9] Farrell M Waibel FWA Carrera I Spattini G Clark L Adams RJ . Musculoskeletal adverse events in dogs receiving bedinvetmab (Librela). Front Vet Sci. (2025) 12:1581490. doi: 10.3389/fvets.2025.1581490 40417367 PMC12100767

[10] Veterinary Medicines Directorate (VMD). Results of in-Depth Assessment of Selected Adverse Events for “Librela Solution for Injection for Dogs” (2026). GOV.UK. Available online at: https://www.gov.uk/government/publications/in-depth-assessment-of-selected-adverse-events-for-librela-solution-for-injection-for-dogs (Accessed July 19, 2026).

[11] Mobasheri A Hanson P Larkin J. Rapidly progressive osteoarthritis (RPOA) in companion animals treated with bedinvetmab (Librela™): an expected pathophysiological phenomenon or a cause for concern? Front Vet Sci. (2025) 12:1640217. doi: 10.3389/fvets.2025.1640217 40948630 PMC12426869

[12] Brunke MW Dewey CW. Commentary: musculoskeletal adverse events in dogs receiving bedinvetmab (Librela). Front Vet Sci. (2025) 12:1628681. doi: 10.3389/fvets.2025.1628681 40740305 PMC12307179

[13] Tiveron C Fasulo L Capsoni S Malerba F Marinelli S Paoletti F . ProNGF\NGF imbalance triggers learning and memory deficits, neurodegeneration and spontaneous epileptic-like discharges in transgenic mice. Cell Death Differ. (2013) 20:1017–30. doi: 10.1038/cdd.2013.22 23538417 PMC3705592

[14] Zampieri N Chao MV. Mechanisms of neurotrophin receptor signalling. Biochem Soc Trans. (2006) 34(Pt 4):607–11. doi: 10.1042/BST0340607 16856873

[15] Nykjaer A Lee R Teng KK Jansen P Madsen P Nielsen MS . Sortilin is essential for proNGF-induced neuronal cell death. Nature. (2004) 427:843–8. doi: 10.1038/nature02319 14985763

[16] Bradshaw RA Pundavela J Biarc J Chalkley RJ Burlingame AL Hondermarck H. NGF and proNGF: regulation of neuronal and neoplastic responses through receptor signaling. Adv Biol Regul. (2015) 58:16–27. doi: 10.1016/j.jbior.2014.11.003 25491371 PMC4426037

[17] Ioannou MS Fahnestock M. ProNGF, but not NGF, switches from neurotrophic to apoptotic activity in response to reductions in TrkA receptor levels. Int J Mol Sci. (2017) 18:599. doi: 10.3390/ijms18030599 28282920 PMC5372615

[18] Kropf E Fahnestock M. Effects of reactive oxygen and nitrogen species on TrkA expression and signalling: implications for proNGF in aging and Alzheimer's disease. Cells. (2021) 10:1983. doi: 10.3390/cells10081983 34440751 PMC8392605

[19] Kropf E Shekari A Jaberi S Puri A Wu C Fahnestock M. Age-induced nitrative stress decreases retrograde transport of proNGF via TrkA and increases proNGF retrograde transport and neurodegeneration via p75NTR. Front Mol Neurosci. (2023) 16:1241420. doi: 10.3389/fnmol.2023.1241420 38025269 PMC10679388

[20] Birder L de Groat W Mills I Morrison J Thor K Drake M. Neural control of the lower urinary tract: peripheral and spinal mechanisms. Neurourol Urodyn. (2010) 29:128–39. doi: 10.1002/nau.20837 20025024 PMC2910109

[21] de Groat WC Griffiths D Yoshimura N. Neural control of the lower urinary tract. Compr Physiol. (2015) 5:327–96. doi: 10.1002/j.2040-4603.2015.tb00596.x 25589273 PMC4480926

[22] Yoshimura N Jeong JY Kim DK Chancellor MB. Integrated physiology of the lower urinary tract. In:Corcos J Ginsberg D Karsenty G, editors. Textbook of the Neurogenic Bladder. 3rd edn. Boca Raton, FL: CRC Press (2015). p. 33–48. doi: 10.1201/b18585-3

[23] de Groat WC Yoshimura N. Afferent nerve regulation of bladder function in health and disease. Handb Exp Pharmacol. (2009) 194:91–138. doi: 10.1007/978-3-540-79090-7_4 19655106 PMC3383010

[24] Schnegelsberg B Sun T-T Cain G Bhattacharya A Nunn PA Ford APDW . Overexpression of NGF in mouse urothelium leads to neuronal hyperinnervation, pelvic sensitivity, and changes in urinary bladder function. Am J Physiol Regul Integr Comp Physiol. (2010) 298:R534–47. doi: 10.1152/ajpregu.00367.2009 20032263 PMC2838659

[25] Arms L Vizzard MA. Neuropeptides in lower urinary tract function. Handb Exp Pharmacol. (2011) 202:395–423. doi: 10.1007/978-3-642-16499-6_19 21290237 PMC4040464

[26] Frias B Lopes T Pinto R Cruz F Cruz CD. Neurotrophins in the lower urinary tract: becoming of age. Curr Neuropharmacol. (2011) 9:553–8. doi: 10.2174/157015911798376253 22654715 PMC3263451

[27] Schnitzer TJ Easton R Pang S Levinson DJ Pixton G Viktrup L . Effect of tanezumab on joint pain, physical function, and patient global assessment of osteoarthritis among patients with osteoarthritis of the hip or knee: a randomized clinical trial. JAMA. (2019) 322:37–48. doi: 10.1001/jama.2019.8044 31265100 PMC6613301

[28] LaBranche TP Bendele AM Omura BC Gropp KE Hurst SI Bagi CM . Nerve growth factor inhibition with tanezumab influences weight-bearing and subsequent cartilage damage in the rat medial meniscal tear model. Ann Rheum Dis. (2017) 76:295–302. doi: 10.1136/annrheumdis-2015-208913 27381034 PMC5264211

[29] Argyropoulos M Wynell-Mayow W Johnson O Faroug R Johal KS Deol RS . Charcot neuro-osteoarthropathy: a review of key concepts and an evidence-based surgical management algorithm. Front Clin Diabetes Healthc. (2024) 5:1344359. doi: 10.3389/fcdhc.2024.1344359 39219847 PMC11362032

[30] Tomlinson RE Li Z Li Z Minichiello L Riddle RC Venkatesan A . NGF-TrkA signaling in sensory nerves is required for skeletal adaptation to mechanical loads in mice. Proc Natl Acad Sci USA. (2017) 114:E3632–41. doi: 10.1073/pnas.1701054114 28416686 PMC5422802

[31] Hochberg MC. Serious joint-related adverse events in randomized controlled trials of anti-nerve growth factor monoclonal antibodies. Osteoarthr Cartil. (2015) 23(Suppl. 1):S18–21. doi: 10.1016/j.joca.2014.10.005 25527216

[32] Monteiro BP Lascelles BDX Murrell J Robertson S Steagall PVM Wright B. 2022 WSAVA guidelines for the recognition, assessment and treatment of pain. J Small Anim Pract. (2023) 64:177–254. doi: 10.1111/jsap.13566

[33] Xu M Li Z Thottappillil N Cherief M Zhu M Xing X . Mapping somatosensory afferent circuitry to bone identifies neurotrophic signals required for fracture healing. Science. (2026) 391:eadr9608. doi: 10.1126/science.adr9608 41505527

[34] Behr B Leucht P Longaker MT Quarto N. Fgf-9 is required for angiogenesis and osteogenesis in long bone repair. Proc Natl Acad Sci USA. (2010) 107:11853–8. doi: 10.1073/pnas.1003317107 20547837 PMC2900703

[35] Li Z Meyers CA Chang L Lee S Li Z Tomlinson R . Fracture repair requires TrkA signaling by skeletal sensory nerves. J Clin Invest. (2019) 129:5137–50. doi: 10.1172/JCI128428 31638597 PMC6877307

[36] Rapp AE Kroner J Baur S Schmid F Walmsley A Mottl H . Analgesia via blockade of NGF/TrkA signaling does not influence fracture healing in mice. J Orthop Res. (2015) 33:1235–41. doi: 10.1002/jor.22892 25876530

[37] Cruz CD Cruz F. Spinal cord injury and bladder dysfunction: new ideas about an old problem. ScientificWorldJournal. (2011) 11:214–34. doi: 10.1100/tsw.2011.26 21258763 PMC5720001

[38] Kordass U Carlson R Stein VM Tipold A. Measurements of C-reactive protein (CRP) and nerve-growth-factor (NGF) concentrations in serum and urine samples of dogs with neurologic disorders. BMC Vet Res. (2016) 12:7. doi: 10.1186/s12917-015-0628-x 26746899 PMC4706657

[39] Montagne A Barnes SR Sweeney MD Halliday MR Sagare AP Zhao Z . Blood-brain barrier breakdown in the aging human hippocampus. Neuron. (2015) 85:296–302. doi: 10.1016/j.neuron.2014.12.032 25611508 PMC4350773

[40] Morita T Mizutani Y Sawada M Shimada A. Immunohistochemical and ultrastructural findings related to the blood–brain barrier in the blood vessels of the cerebral white matter in aged dogs. J Comp Pathol. (2005) 133:14–22. doi: 10.1016/j.jcpa.2005.01.001 15899493

[41] Bassalo D Matthews SG Bloise E. The canine blood-brain barrier in health and disease: focus on brain protection. Vet Q. (2025) 45:12–32. doi: 10.1080/01652176.2025.2450041 39791202 PMC11727060

[42] Merbl Y Mondrus E Hanael E Shamir MH. Blood–brain barrier permeability changes in dogs with suspected canine cognitive dysfunction using magnetic resonance imaging subtraction enhancement analysis. Front Vet Sci. (2025) 12:1572286. doi: 10.3389/fvets.2025.1572286 40433457 PMC12106434

[43] Enomoto M de Castro N Hash J Thomson A Nakanishi-Hester A Perry E . Prevalence of radiographic appendicular osteoarthritis and associated clinical signs in young dogs. Sci Rep. (2024) 14:2827. doi: 10.1038/s41598-024-52324-9 38310147 PMC10838335

[44] Zhang Y Lee DHS. Sink hypothesis and therapeutic strategies for attenuating Aβ levels. Neuroscientist. (2011) 17:163–73. doi: 10.1177/1073858410381532 21330304

[45] Dewey CW Brunke MW. Dysmetabolism of the nerve growth factor pathway in the aging brain plays a pivotal role in cognitive decline. J Am Vet Med Assoc. (2026) 264:471–5. doi: 10.2460/javma.25.09.0578 41349274

[46] Cano A Fonseca E Ettcheto M Sánchez-López E de Rojas I Alonso-Lana S . Epilepsy in neurodegenerative diseases: related drugs and molecular pathways. Pharmaceuticals. (2021) 14:1057. doi: 10.3390/ph14101057 34681281 PMC8538968

[47] Averill S McMahon SB Clary DO Reichardt LF Priestley JV. Immunocytochemical localization of TrkA receptors in chemically identified subgroups of adult rat sensory neurons. Eur J Neurosci. (1995) 7:1484–94. doi: 10.1111/j.1460-9568.1995.tb01143.x 7551174 PMC2758238

[48] European Medicines Agency (EMA). Lenivia (Izenivetmab): Veterinary Medicinal Product Information (2025). Available online at: https://medicines.health.europa.eu/veterinary/en/600001881644 (Accessed July 19, 2026).

[49] European Medicines Agency (EMA). Lenivia: Summary of Opinion (Initial Authorisation). EMA/CVMP/308157/2025 (2025). Available online at: https://www.ema.europa.eu/en/documents/smop-initial/cvmp-summary-positive-opinion-lenivia_en.pdf (Accessed July 19, 2026).

[50] Health Canada. Lenivia (Izenivetmab), DIN 02561816: Product Monograph/Veterinary Labelling (2025). Available online at: https://health-products.canada.ca/dpd-bdpp/info?code=106474&lang=eng (Accessed July 19, 2026).

[51] Belanger P Butler P Butt M Bhatt S Foote S Shelton D . From the cover: evaluation of the effects of tanezumab, a monoclonal antibody against nerve growth factor, on the sympathetic nervous system in adult cynomolgus monkeys (*Macaca fascicularis*): a stereologic, histomorphologic, and cardiofunctional assessment. Toxicol Sci. (2017) 158:319–33. doi: 10.1093/toxsci/kfx089 28525647 PMC5837719

